# A Function for the hnRNP A1/A2 Proteins in Transcription Elongation

**DOI:** 10.1371/journal.pone.0126654

**Published:** 2015-05-26

**Authors:** Bruno Lemieux, Marco Blanchette, Anne Monette, Andrew J. Mouland, Raymund J. Wellinger, Benoit Chabot

**Affiliations:** 1 RNA group and Department of Microbiology and Infectious Diseases, Faculty of Medicine and Health Sciences, Université de Sherbrooke, Sherbrooke, Quebec, Canada; 2 Stowers Institute for Medical Research, Kansas City, Missouri, United States of America; 3 Lady Davis Institute for Medical Research-Sir Mortimer B. Davis Jewish General Hospital, McGill University, Montreal, Quebec, Canada; Florida Atlantic University, UNITED STATES

## Abstract

The hnRNP A1 and A2 proteins regulate processes such as alternative pre-mRNA splicing and mRNA stability. Here, we report that a reduction in the levels of hnRNP A1 and A2 by RNA interference or their cytoplasmic retention by osmotic stress drastically increases the transcription of a reporter gene. Based on previous work, we propose that this effect may be linked to a decrease in the activity of the transcription elongation factor P-TEFb. Consistent with this hypothesis, the transcription of the reporter gene was stimulated when the catalytic component of P-TEFb, CDK9, was inhibited with DRB. While low levels of A1/A2 stimulated the association of RNA polymerase II with the reporter gene, they also increased the association of CDK9 with the repressor 7SK RNA, and compromised the recovery of promoter-distal transcription on the *Kitlg* gene after the release of pausing. Transcriptome analysis revealed that more than 50% of the genes whose expression was affected by the siRNA-mediated depletion of A1/A2 were also affected by DRB. RNA polymerase II-chromatin immunoprecipitation assays on DRB-treated and A1/A2-depleted cells identified a common set of repressed genes displaying increased occupancy of polymerases at promoter-proximal locations, consistent with pausing. Overall, our results suggest that lowering the levels of hnRNP A1/A2 elicits defective transcription elongation on a fraction of P-TEFb-dependent genes, hence favoring the transcription of P-TEFb-independent genes.

## Introduction

The majority of mammalian genes contain introns that are removed by RNA splicing during or after transcription. While transcription and splicing can be studied independently, these processes are coordinated for optimal gene expression [1–4]. The CTD domain of the large RNA polymerase II subunit permits the coupling of transcription with splicing and other steps of RNA maturation. Phosphorylation of heptad repeats in the CTD triggers interactions with a variety of RNA maturation factors including 5’ capping, splicing, polyadenylation and mRNA export components [5, 6]. TFIIH catalyzes Ser5 phosphorylation on the CTD repeats which facilitates promoter clearance and the interaction with capping factors [1]. In contrast, CDK9, a component of P-TEFb that phosphorylates Ser2 on the CTD repeats, confers a more productive elongation mode [7]. A portion of P-TEFb associates with the repressor 7SK RNA complex [8], and hnRNP A1 and A2 proteins have been proposed to associate with 7SK RNA to control the release of P-TEFb via competitive binding [9, 10].

Transcription can also impact alternative splicing decisions. In mammals, differences in the composition of transcription complexes, chromatin components or a slow RNA polymerase can affect splice site selection [2, 11, 12]. Even in fission yeast, RNA polymerase complexes can be critical for splicing [13]. Conversely, components of the RNA processing machinery can also affect transcription. For example, TAT-SF1 and SKIP, the mammalian homologues of the yeast splicing factors CuB and Prp45, have been implicated in transcription elongation [14, 15]. Likewise, while SR proteins are recruited to the CTD of RNA polymerase II from where they are loaded onto nascent RNA to modulate splicing decisions [16], the SR protein SC35 helps to recruit P-TEFb to elongating transcription complexes [17]. The roles of additional splicing regulators in elongation and other steps of transcription has not yet been systematically investigated.

hnRNP proteins represent a diverse and abundant group of mammalian splicing modulators [18]. In addition to their role in splice site selection, the hnRNP A1 and A2 proteins have been implicated in a variety of cellular functions including mRNA stability [19], mRNA transport [20], miRNA maturation [21] and telomere biogenesis [22–25]. Some hnRNP proteins have also been associated with transcriptional control. For instance, hnRNP K and A1 can interact with promoters to enhance and repress transcription of *c-myc* and *γ-fibrinogen*, respectively [26, 27]. hnRNP U associates with actin to regulate transcription [28, 29]. hnRNP A2 can interact with RNA polymerase II [16], and hnRNP A1 is part of complexes containing the transcription initiation factors TFIIA-55 and TFIIE-34 [30]. Most importantly for our findings here, there is evidence that hnRNP proteins A1 and A2, associate transiently with the small RNA 7SK [9, 10, 31, 32]. Although 7SK regulates the activity of the transcription elongation complex P-TEFb [33, 34], a role for hnRNP proteins in this latter process has not yet been reported.

Here we document a role for the mammalian hnRNP A1 and A2 proteins in transcription elongation. Specifically, the strong stimulation in the transcription of a reporter gene elicited by the RNAi-mediated knockdown of hnRNP A1/A2 led us to hypothesize that this stimulation might be a consequence of a global problem in transcription elongation. In accord with this model, depleting hnRNP A1/A2 reproduced many of the specific and global features of the transcriptional responses associated with P-TEFb inhibition. Overall, our results are consistent with the view that hnRNP A1/A2 proteins facilitate gene expression by activating P-TEFb.

## Material and Methods

### Cell culture

HCT116 and WI38VA13 cells were obtained from the American Type Culture Collection (Manassas, VA). Cells were grown in Dulbecco’s modified Eagle’s medium (DMEM) supplemented with 10% fetal bovine serum (FBS). Cells were treated with the p38MAPK inhibitor SB203580 (Sigma-Aldrich), 5,6-Dichloro-1-β-D-ribofuranosylbenzimidazole (DRB; Calbiochem), actinomycin D (actD; Sigma-Aldrich), cycloheximide (Sigma-Aldrich), or D-sorbitol (Sigma-Aldrich) in DMEM supplemented with 10% FBS.

Osmotic shock treatment with sorbitol was performed by incubating cells in complete DMEM supplemented with 600 mM sorbitol for 1 hour. Cells were then washed with PBS and left in fresh DMEM with 10% FBS for the indicated period of time. When using a specific compound during an osmotic shock, all media were supplemented with the compound during the experiment and cells were pretreated for 2 hours (SB203580 at 8 μg/ml, actinomycin D (actD) at 5 μg/ml) or 1 hour (cycloheximide at 1 or 10 μg/ml). In the case of the combination actD + siRNA, cells were treated with 1 μg/ml of actD for the last 24 hours of each period that was investigated.

Cells were transfected with pCMVmycUP1 using Lipofectamine 2000 (Invitrogen). Two days after transfection, cells were reseeded at a lower density and 800 μg/ml of Geneticin (Invitrogen) was added for positive selection of stably transfected cells. Clonal zones were individually reseeded in 24-wells plate and screened for protein expression. For siRNA transfections, we used RNA oligos purchased from Dharmacon Research, Inc (Lafayette, CO). SiRNA sequences are listed in Table A in S1 File. The day before transfection, exponentially growing cells were trypsinized and seeded into six-well plates. Transfections were performed with 40 nM of total siRNA using Lipofectamine 2000 (Invitrogen) according to manufacturer’s instructions.

### Plasmid

pCMVmycUP1 was constructed by ligating the HindIII-EcoRI fragment of a myc-tagged UP1 cDNA [24] into the HindIII and EcoRI sites in pcDNA3.1 (Invitrogen).

### Western Blotting

Whole cell extract was prepared by lysing cells in Laemmli sample buffer (1X = 10% glycerol, 5% β-mercaptoethanol, 2.3% SDS, 62.5 mM Tris-HCl (pH 6.8), and 0.1% bromophenol blue). Equal amounts of total protein was loaded onto 10% SDS-PAGE and transferred to nitrocellulose membranes. Blots were probed with primary antibodies: anti-A1/A2, anti-myc (OP10, EMD Biosciences Inc) or anti-α-tubulin (2144S, Cell Signaling) antibodies, and revealed with peroxidase-conjugated secondary antibodies and ECL detection reagent (Amersham).

### RT-PCR

Total RNA was prepared with TRIzol reagent following manufacturer’s instructions (Invitrogen). RT reactions were performed with MMuLV reverse transcriptase on 1 μg of total RNA using a T20VN tailed variant (RT101) mixed with random hexamers. For quantification of 7SK RNA, RT102_7SK and RT101 primers were used. Primers were designed against sequences of the indicated gene using Primer3 online resource [35]. Primer sequences are listed in Table B in S1 File. Semi-quantitative RT-PCR (sqRT-PCR) products were resolved on 1% agarose gel and quantified using Quantity One software (Bio-Rad). Fold enrichment was normalized with standard curves from a cDNA sample and the level of ACTB mRNA products. Quantitative RT-PCR (qRT-PCR) was performed on a Eppendorf Mastercycler ep realplex Thermal Cycler using qRT-PCR primers listed in Table B in S1 File and using the FastStart Universal SYBR green master (ROX) (Roche).

### Immunostaining

HCT116 cells were seeded onto glass coverslips and grown at 37°C in a humidified atmosphere containing 5% CO_2_ in complete growth medium consisting of Dulbecco’s modified Eagle’s medium (DMEM) supplemented with 10% fetal bovine serum (FBS). Twenty-four hours later, when cells were approximately 80% confluent, the medium was replaced with complete growth medium or with complete growth medium + 600 mM sorbitol and cells were allowed to continue growth for 1 hour prior to being collected for analysis as described [36]. The immunofluorescence analysis has recently been described elsewhere [37]. Briefly, cells were fixed using 4% paraformaldehyde, permeabilized with 0.2% Triton-X100 and blocked with 10% dry milk in PBS before being incubated with the primary antibodies. These are mouse monoclonal and rabbit polyclonal anti-hnRNP A2 and anti-hnRNP A1 respectively, and have been provided by William Rigby (Dartmouth Medical School, NH, USA). Secondary fluorophore-conjugated antisera (Alexa Fluor 488 and 564) were obtained from Molecular Probes at Invitrogen. Images were acquired using laser scanning confocal microscopy. Confocal laser microscopy was performed on a LSM5 Pascal (Carl-Zeiss) equipped with a Plan-Apochromat 63x oil immersion objective and an Ar/Kr laser. Alexa Fluor 594 and 488 antibodies were scanned using excitation wavelengths of 543 nm and 488 nm, and emission spectra were filtered with 560–615 nm and 505–530 nm bandpass filters respectively and sequentially.

### RNA polymerase II chromatin immunoprecipitation (ChIP) assays

ChIP assays are performed essentially as described in Svotelis et al. (2009) [38]. Briefly, after PBS wash, treated cells were cross-linked with a 1% formaldehyde/PBS solution for 10 min at room temperature. Cells were washed twice with cold PBS and harvested in buffer A (100 mM Tris-HCl at pH 9.4, 1 mM PMSF, 10 mM DTT, 2 μg/ml leupeptin and 2 μg/ml aprotinin) and incubated at 30°C for 20 min. Cells are further washed with buffer I (0.25% Triton, 10 mM EDTA, 0.5 mM EGTA, 10 mM HEPES-KOH at pH 6.5, 1 mM PMSF, 2 μg/ml leupeptin and 2 μg/ml aprotinin) and buffer II (200 mM NaCl, 1 mM EDTA, 0.5 mM EGTA, 10 mM HEPES-KOH at pH 6.5, 1 mM PMSF, 2 μg/ml leupeptin and 2 μg/ml aprotinin). Lysis was performed in SDS lysis buffer (1% SDS, 10 mM EDTA, 50 mM Tris pH 8.1, 1 mM PMSF, 2 μg/ml leupeptin and 2 μg/ml aprotinin) for 10 min on ice. Samples were diluted in IP dilution buffer (0.01% SDS, 1.1% Triton, 1.2 mM EDTA, 16.7 mM Tris-HCl at pH 8.1, 167 mM NaCl, 1 mM PMSF, 2 μg/ml leupeptin and 2 μg/ml aprotinin) and sonicated to generate DNA fragments < 1 kb. Samples were precleared for 2 h with 30 μl of 50% protein-A sepharose CL4B (GE) (pre-blocked with 2 μg salmon sperm DNA) and 1 mg/ml BSA. Two micrograms of RNA polymerase II antibody H-224 (Santa-Cruz, sc-9001X) was added to the sample and incubated overnight at 4°C. Immunocomplexes were recovered using 30 μl of fresh 50% proteinA sepharose CL4B blocked beads. Beads were washed twice with TSE-150 (0.1% SDS, 1% Triton, 2 mM EDTA, 20 mM Tris-HCl at pH 8.1, 150 mM NaCl), three time with TSE-500 (0.1% SDS, 1% Triton, 2 mM EDTA, 20 mM Tris-HCl at pH 8.1, 500 mM NaCl), twice with LiCl detergent (0.25 M LiCl, 1% NP-40, 1% DOC, 1 mM EDTA, 10 mM Tris-HCl at pH 8.1) and finally twice with TE1X. Immunocomplexes were eluted for 20 min at 65°C with elution buffer (1% SDS, 0.1 M NaHCO_3_) and cross-linking was reversed by incubating overnight at 65°C. DNA was purified using QIAquick PCR purification kit and quantified by quantitative real-time PCR using primers listed in Table C in S1 File. Input DNA and the no antibody sample were used for normalization. Results are from three independent experiments.

For the Chip-seq experiment, the DNA samples from the pol II chromatin-immunopurified samples were processed into an Illumina compatible library using a ChIP-Seq kit following the manufacturer's protocol (Illumina). The library was then sequenced for 40 cycles on the Illumina GAIIx platform following the manufacturer's recommendations (Illumina). The 40-nucleotide (nt) reads from the sequencer Fastq files were then aligned to the human genome (hg19) with bowtie, using the options-S-a-p6-m1-n2-best-strata, and the resulting alignment were then converted to binary sorted alignments in BAM format using SamTools [39]. A total of 9.2 M, 9.7 M and 9.5 M 40-nt reads were generated for the control, DRB- and siA1/A2-treated samples, respectively, and 64.3% (5.9 M), 69.1% (6.7 M) and 66.3% (6.3 M) of the reads were uniquely aligned to the human genome for the respective library.

### 7SK RNA recovery assay

7SK RNA immunoprecipitation was performed as described [40] using HCT116 cells left untreated, siRNA-treated (72 h) or treated with 100 μM DRB (6 h). Briefly, 4 x 10^6^ cells were lysed on ice for 10 min in buffer A (20 mM HEPES-KOH (pH 7.8), 10 mM KCl, 0.2 mM EDTA and 0.5% Nonidet P-40). Supernatants of lysates were pre-cleared with protein-A sepharose CL-4B precoated with BSA and yeast tRNA and divided into two aliquots. Each aliquot was incubated with either no antibody or 1 μg of anti-CDK9 (sc-484) overnight at 4°C in the presence of BSA and then with 15 μl of precoated beads for 2 hours. Beads were washed five times with buffer B (20 mM HEPES-KOH (pH 7.8), 100 mM KCl, 0.2 mM EDTA and 0.5% Nonidet P-40). RNA were extracted by TRIzol (Invitrogen) and analyzed by qRT-PCR using a specific tailed oligo to 7SK for the RT step. Data were normalized to input amount of 7SK RNA.

### Transcriptome sequencing

Total RNA from cells treated with DRB, with siRNAs targeting hnRNP A1 and A2 or not-treated (control) was purified using the RNAeasy mini kit (Qiagen). A total of 10 μg of RNA was used to generate an TruSeq library using the manufacturer’s protocol (Illumina). The library was then sequenced for 51 cycles on a Illumina HiSeq-2500 platform following the manufacturer’s recommendations (Illumina). The 51 nt reads from the sequencer Fastq files were then aligned to the human genome (hg19) with TopHat v2.0.10, using the options-p6—no-coverage-search with a transcriptome index produced from the Ensemble GRCh37 release 73 gene build.

A total of 122.1, 142.4 M and 100.9 M 51-nt reads were generated for the control, DRB- and siA1/A2-treated samples, respectively. 76.3%, 65.9% and 67.7% of reads were successfully aligned and 69.8% (85.2 M), 59.0% (84.1 M) and 58.3% (58.9 M) of the total reads were uniquely aligned to the human genome for each of the respective library. RPKM (Reads per kilobase of transcript per million mapped reads) per genes were then produced by counting the number of uniquely mappable reads that fell within exonic region for all the genes annotated in the Ensembl GRCh37 release 73 build. The total length of exonic regions were used to normalize read counts per gene.

### Transcription elongation assay

The assay was performed as described in Singh and Padgett [41]. HCT116 cells transfected for 72 hours with siRNA against hnRNP A1 and A2 (siA1_6_ + siA2_1_) or with siLuc as control were then treated with 100 μM DRB for 3 hours, washed with PBS and incubated in fresh medium before total RNA was extracted at each time point using TRIzol (Invitrogen). Changes in the level of pre-mRNA at each position along the *Kitlg* gene were quantified by qRT-PCR using primers listed in Table C in S1 File. Values obtained are normalized relative to the average mRNA level of a set of reference genes (*ACTB*, *B2M*, *GAPDH* and *RPL13A*; Table B in S1 File) for each time point. Each assay represents a biological triplicate.

## Results

### The depletion of nuclear hnRNP A1 and A2 stimulates the expression of a reporter gene

**Fig 1:**
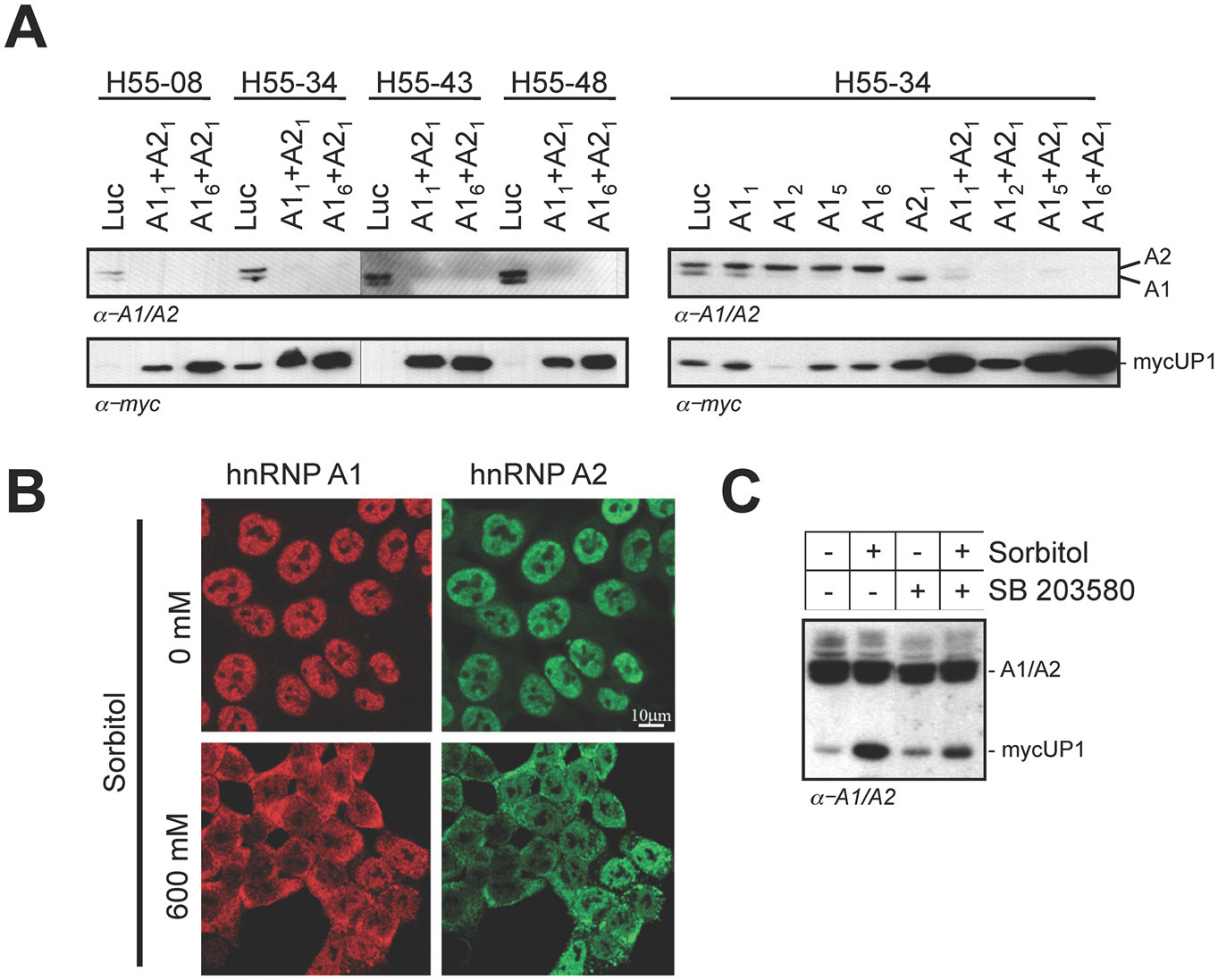
mycUP1 expression is stimulated by the depletion of nuclear of hnRNP A1 and A2. **A**, Western analysis of A1, A2 and mycUP1 from four selected HCT116 clones engineered to express a transfected myc-tagged mouse UP1. Stable transformants were transfected with various siRNA mixtures and proteins were extracted 72 hours later. Only siRNA A1_2_ also targets the mouse mycUP1. **B**, Cytoplasmic accumulation of hnRNP A1 and hnRNP A2 in HCT116 cells upon osmotic shock. HCT116 cells growing on glass coverslips were left untreated (0 mM) or were exposed to 600 mM sorbitol. After 1 hour, cells were fixed and immunostained with anti-hnRNP A1 and anti-hnRNP A2 antibodies. **C**, Sorbitol stimulates mycUP1 expression in a p38 kinase-dependent manner. The p38 kinase inhibitor SB203580 was added to H55-34 cells for 2 hours (8 μg/ml) before treating cells with 600 mM sorbitol for 1 hour. Cells were then washed with PBS and incubated in complete media for 24 hours. Endogenous A1/A2 and mycUP1 proteins were detected simultaneously using the anti-A1/A2 antibody.

For an unrelated project, we produced clonal derivatives of the human colon cancer cell line HCT116 expressing a reporter gene made from a truncated and myc-tagged version of the mouse hnRNP A1 cDNA (mycUP1) under the control of the CMV promoter. As judged by western analysis, the level of mycUP1 protein in all four stable clones was low and in most cases undetectable (Fig 1A, lanes Luc; α-myc panel). However, the siRNA-mediated depletion of the human hnRNP A1 and A2 proteins provoked a marked increase in mycUP1 protein level (Fig 1A). This stimulation was observed with a different combination of siRNAs against A1 and A2, but not when A1 and A2 were targeted individually, possibly because of functional redundancy between the two proteins, and because a reduction in the expression of one protein appears to increase the expression of the other (Fig 1A, right panels).

Another way of depleting hnRNP A1 from the nucleus is through osmotic shock, as this treatment provokes the cytoplasmic retention of hnRNP A1 [36, 42–44]. Osmotic shock with sorbitol similarly affects the localization of hnRNP A2 (Fig 1B). Sorbitol stimulated the production of mycUP1 protein (Fig 1C; in this case the mycUP1 protein was revealed by the anti-hnRNP A1/A2 antibody). The cytoplasmic accumulation of A1 occurs because osmotic stress activates the MEK_3/6_-p38 kinase pathway that stimulates the phosphorylation of A1 through the Mnk kinases [36, 43]. Consistent with this mechanism, inhibiting the p38 kinase with SB203580 reduced the impact of sorbitol on the production of mycUP1 protein (Fig 1C). The effect observed on the reporter gene was not restricted to HCT116 cells since decreasing the nuclear levels of A1 and A2 by osmotic shock or RNA interference also stimulated mycUP1 protein levels in WI38VA13 cells (Fig A in S1 File).

**Fig 2:**
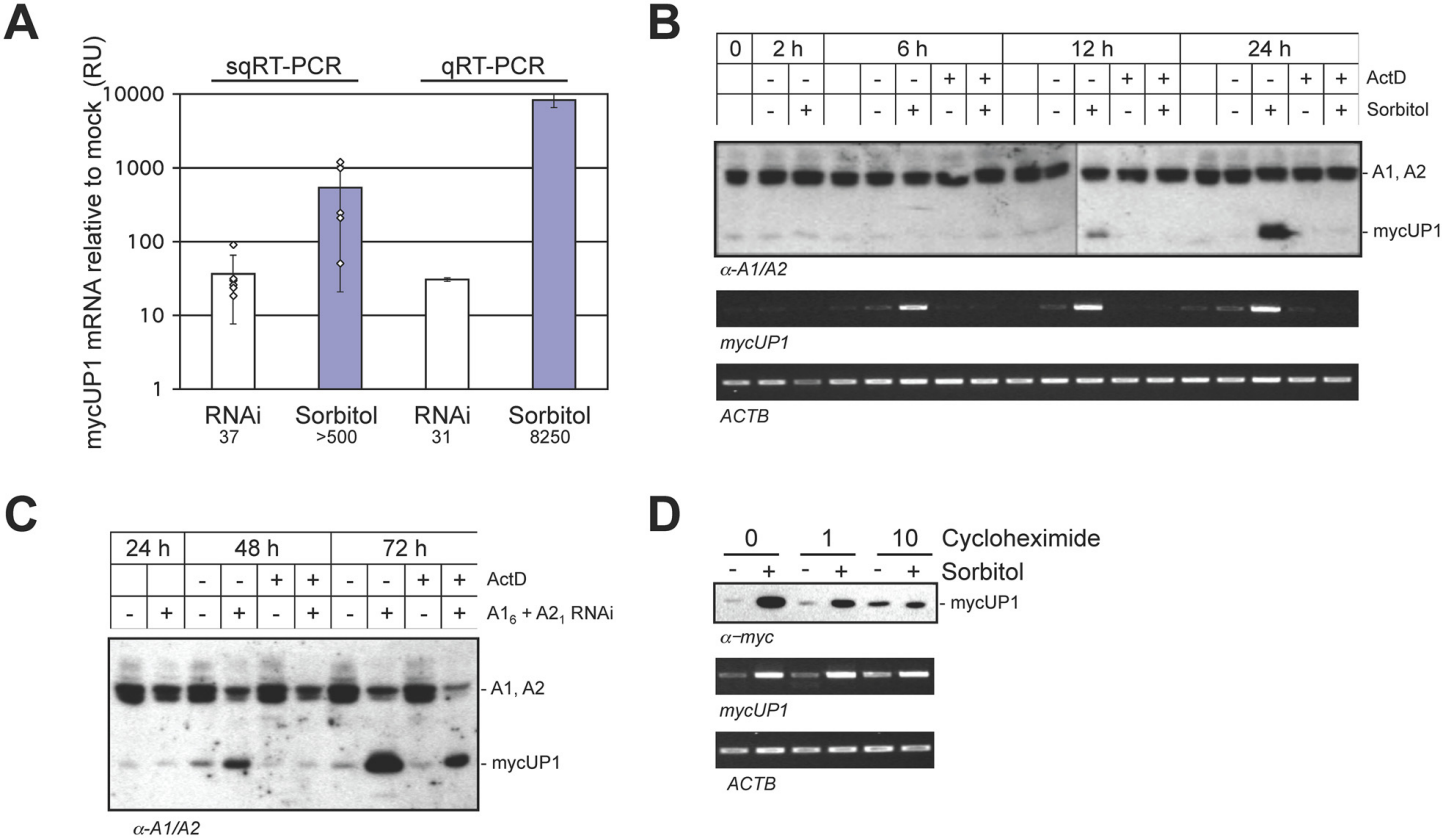
The depletion of nuclear hnRNP A1 and A2 stimulates the transcription of mycUP1. **A**, Steady-state levels of mycUP1 transcripts measured by semi-quantitative or real-time quantitative RT-PCR (sqRT-PCR or qRT-PCR). At least 5 independent depletion experiments (A1_6_+A2_1_ vs Luc) and sorbitol treatments (0 or 600 mM) were analyzed by sqRT-PCR (examples are shown in Fig B in S1 File). Fold increases are represented by white diamonds over an averaged logarithmic histogram. Numbers below the histograms indicate averaged fold stimulation in mycUP1 transcripts. **B**, Actinomycin D (ActD) abrogates the increase in mycUP1 protein and RNA expression following osmotic shock. The top panel represents a western with the A1/A2 antibody and the results of sqRT-PCR for mycUP1 and actin (ACTB) are shown below. **C**, ActD abrogates the increase in mycUP1 protein expression associated with the knockdown of A1 and A2. Western analysis of mycUP1 protein expression is shown at different times after siRNA treatment with or without ActD for the last 24 hours of each period. **D**, HCT116 cells expressing mycUP1 were treated with 1 or 10 μg/ml of cycloheximide 1 hour prior to sorbitol treatment. The mycUP1 protein and RNA levels are shown. Duplicate experiments with quantitated mycUP1 transcript levels are provided in Fig C in S1 File.

The above assays were performed several times with similar results. Although the level of stimulation is striking, it is difficult to estimate because the mycUP1 protein is not always detected in the controls. We performed RT-PCR to determine if mycUP1 mRNA levels also increased, and if so, to what extent, Semi-quantitative RT-PCR assays indicate that the steady-state levels of mycUP1 transcripts in the HCT116-derived H55-34 cell line respectively increased by 37- and more than 500-fold after treatment with siA1/siA2 and sorbitol, respectively (Fig 2A and Fig B in S1 File). Quantitative RT-PCR (qRT-PCR) confirmed this assessment (30- and 8250-fold, for siA1/A2 and sorbitol, respectively; Fig 2A).

Because mycUP1 is related to hnRNP A1 but lacks its glycine-rich C-terminal portion, we considered the possibility that stimulation may be part of a feedback loop that stabilizes the mycUP1 mRNA. Consistent with the view that stimulation is not caused by increased mRNA stability, blocking transcription with actinomycin D abrogated the increases in mycUP1 RNA and mycUP1 protein induced by sorbitol and by the knockdown of A1/A2 (Fig 2B and 2C).

The depletion of nuclear A1/A2 may also stimulate mycUP1 expression indirectly. For example, as hnRNP A1 and A2 regulate the alternative splicing of many genes [45, 46], the decrease in the level of A1/A2 proteins may produce splice variants that in turn stimulate the expression of mycUP1. If this were the case, producing such variants would require protein synthesis. We used the translation inhibitor cycloheximide to address this possibility. As shown in Fig 2D and Fig C in S1 File, while cycloheximide abolished the sorbitol-induced mycUP1 protein expression, it did not severely compromise the increase in mycUP1 RNA expression, indicating that this stimulation did not require *de novo* protein synthesis, We did not test the impact of cycloheximide on the stimulation elicited by the depletion of A1/A2 by RNAi because this depletion requires 72 hours to take full effect, and cycloheximide will kill cells if applied for such a long period. Overall, our results suggest that the depletion of nuclear A1/A2 affects transcription of the mycUP1 reporter in a fashion that is distinct from its known roles in splicing regulation and from RNA stability issues. Although we have not specifically tested if the RNAi-mediated knockdown of A1/A2 depletes A1/A2 from the nucleus, the bulk of hnRNP A1/A2 proteins are mostly nuclear (Fig 2), and our knockdown efficiency is typically superior to 75% for both A1 and A2 (Fig 1). Hence, we assume that the nuclear levels of A1 and A2 are reduced by the siRNA-mediated depletion. We will use the term « nuclear depletion » to refer to the common impact of siRNA-mediated knockdown and sorbitol on A1/A2 levels.

### The depletion of nuclear hnRNP A1 and A2 increases the association of RNA polymerase II on the mycUP1 reporter gene

**Fig 3:**
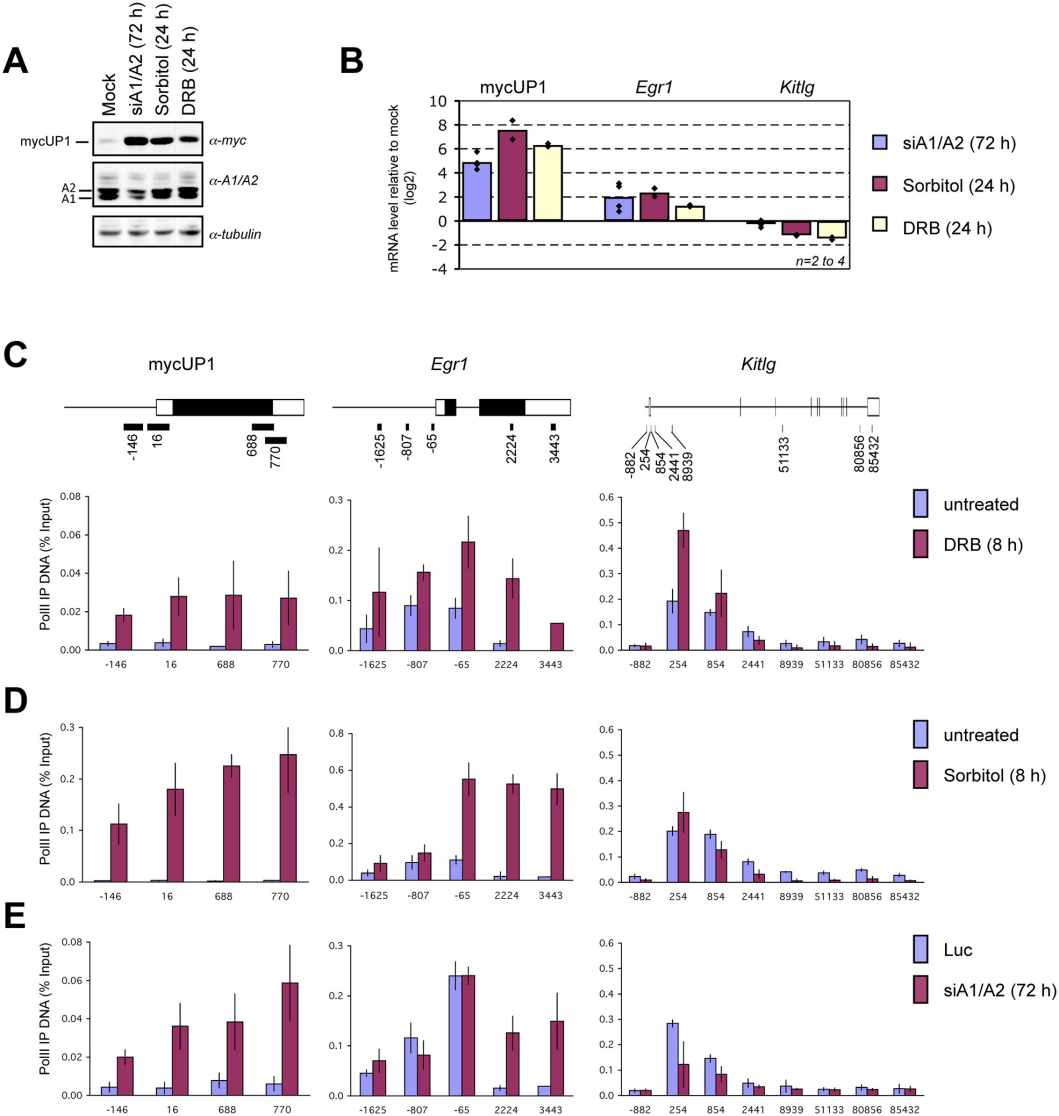
Depleting nuclear hnRNP A1/A2 mimics the effect of the transcription elongation inhibitor DRB. **A**, The P-TEFb kinase CDK9 was inactivated by treating cells with DRB (100 μM) for 24 hours. The impact on mycUP1 protein expression was compared to the impact of a 1 hour treatment with sorbitol (cells harvested 24 hours later), treatment with siA1/A2 for 72 hours or untreated (mock). **B**, Quantitative RT-PCR was used to measure the impact of siA1/A2, sorbitol or DRB on the steady state levels of mycUP1 transcripts from the reporter, and endogenous *Egr1* and *Kitlg* transcripts. Levels are expressed relative to the mock treatment. **C**, Occupancy profiling of RNA polymerase II on the mycUP1, *Egr1*, *Kitlg* and genes. ChIP assays were performed with extracts of H55-34 cells using the pol II antibody H-224. A linear map of each gene locus is provided. Exons are represented by boxes and coding sequences are in black. The location of amplicons used in qPCR is shown below and numbers indicate the relative position of the center of the amplicon relative to the transcription start site. Pol II-ChIP performed on extract produced after DRB treatment (100 μM) for 8 hours. **D**, Pol II-ChIP performed on extract prepared 8 hours following the application of sorbitol for 1 hour. **E**, Pol II/ChIP performed on extract from a 72 hours of RNAi treatment targeting hnRNP A1 and A2. For panels C–E, the DNA recovered after ChIP was quantitated by qPCR using the indicated amplicons. Values are expressed as percentage of input DNA. Error bars indicate standard deviations and are based on three independent experiments.

One way through which hnRNP A1 and A2 may affect transcription is by controlling the release of the transcription elongation factor P-TEFb from its repressor, the 7SK RNA [9, 10]. Indeed, reducing the levels of A1 and A2 prevents the release of P-TEFb from the P-TEFb-HEXIM1-7SK complex *in vitro* [9]. If A1/A2 function at this step, promoter-proximal RNA polymerase II stalling would be predicted to occur on P-TEFb-dependent genes, whereas P-TEFb-independent genes would in principle not be affected. Possibly however, P-TEFb-independent genes such as mycUP1 may experience increased transcription if a general early block of elongation occurs on enough P-TEFb-dependent genes as to lead to more RNA polymerases becoming available. If this proposition is valid, stalling polymerases by pharmacologically inhibiting the P-TEFb kinase CDK9 with 5,6-di-chloro-1-b-D-ribofuranosyl-benzimidazole (DRB) should mimic the effect of depleting A1/A2 and should stimulate the expression of mycUP1. Strikingly, DRB had a strong stimulatory effect on mycUP1 protein and mycUP1 RNA level yielding an increase that was similar in amplitude to osmotic shock and the siRNA-mediated depletion of A1/A2 (Fig 3A and 3B).

Our results suggest that the depletion of nuclear A1/A2 by sorbitol or RNAi may increase the association of RNA polymerase II (pol II) with the mycUP1 gene. To assess pol II occupancy on mycUP1, we performed a chromatin immunoprecipitation (ChIP) assay using quantitative PCR to measure the recovery of associated DNA fragments. We used the endogenous *Egr1* and *Kitlg* genes as controls since their expression respectively increased and decreased when cells were depleted of hnRNP A1/A2, treated with sorbitol or DRB (Fig 3B). DRB, sorbitol and the siRNA-mediated depletion of A1/A2 increased pol II occupancy at all positions tested on mycUP1 and *Egr1* (Fig 3C–3E), consistent with the stimulation in steady-state levels of transcripts from these genes. In contrast, DRB, sorbitol and siA1/A2 did not increase pol II occupancy on *Kitlg*, except at promoter-proximal positions (Fig 3C–3E and see below). Our results therefore suggest that the strong stimulation in mycUP1 expression associated with sorbitol and the siRNA-mediated depletion of A1/A2 is most likely caused by increased pol II transcription. Importantly, these conclusions can be transposed to the *Egr1* gene, suggesting that a decrease in A1/A2 also affects the expression of endogenous genes.

### The siRNA-mediated depletion of A1/A2 increases the interaction of CDK9 with 7SK

**Fig 4:**
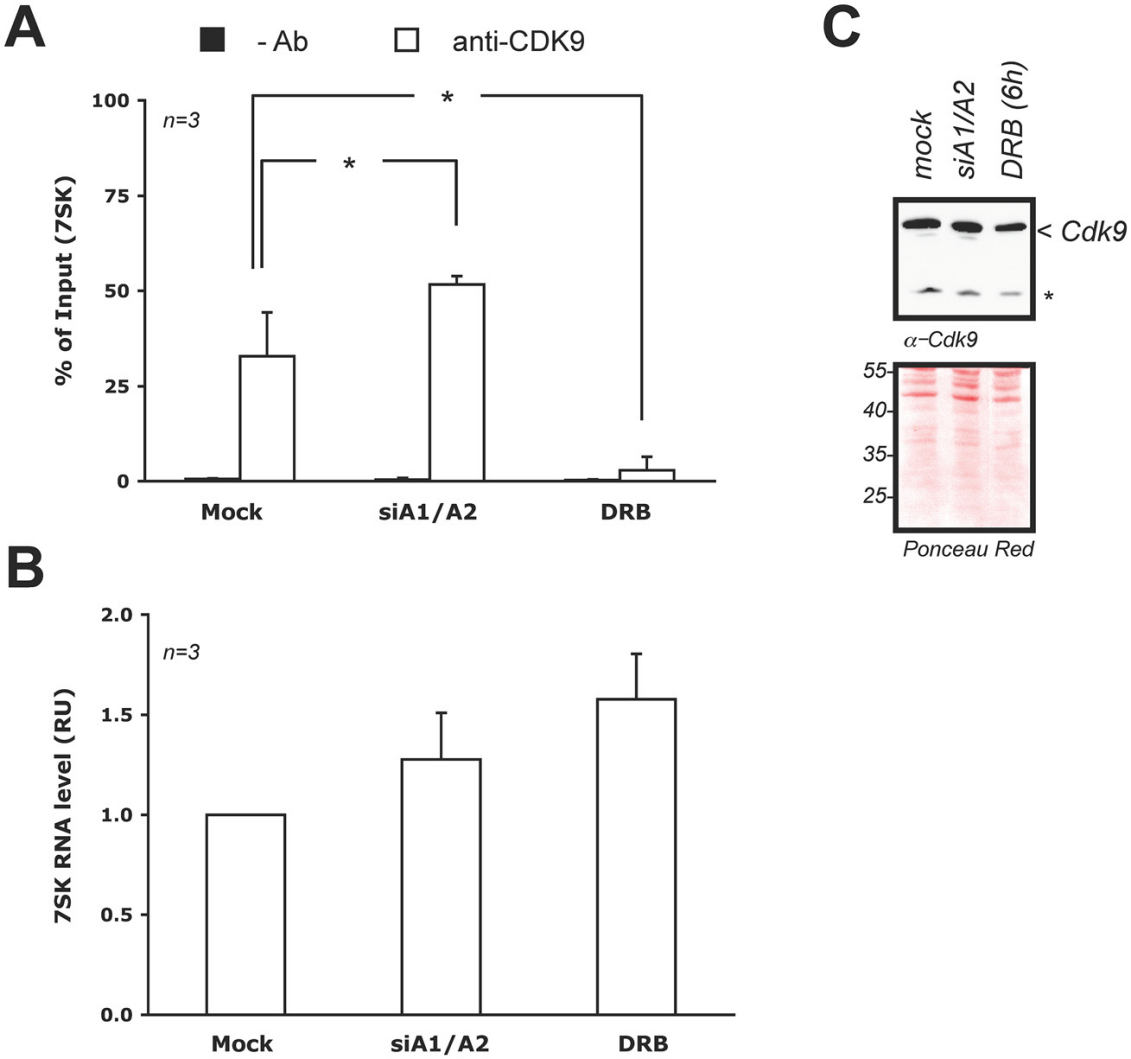
Linking hnRNP A1/A2 with P-TEFb. **A**, Association of 7SK with P-TEFb. The association between 7SK snRNA and CDK9 was tested 72 hours post-transfection with the A1/A2 siRNAs, or 6 hours after treatment with DRB (100 μM). RNA immunoprecipitation (RNA-IP) with anti-CDK9 or without antibody as control were performed and the recovered RNA was quantitated by qRT-PCR with primers specific for 7SK. Data are presented as percent of input 7SK and are averages of 3 experiments with error bars representing SD. *: *p*<0.05, two tailed, two sample equal variance, Student’s t-test. **B**, Relative amount of 7SK RNA in extracts used for RNA-IP experiments quantified by qRT-PCR. The amounts were normalized for *ACTB*, *B2M* and mock treatment. **C**, The level of CDK9 in input samples was evaluated by immunublotting using an anti-CDK9 antibody and Ponceau staining of the membrane to show equivalent loading. The presence of a non-specific band reacting with the anti-CDK9 antibody is indicated by an asterisk (*).

The similar impact of DRB on the expression of mycUP1 and on RNA polymerase II occupancy suggests the possibility that a transcription elongation defect may be occurring when A1/A2 are depleted from the nucleus in HCT116 cells. While DRB directly inhibits CDK9 enzymatic activity [47], circumstantial evidence suggests that hnRNP A1 and A2 may affect transcription elongation by controlling the release of the transcription elongation factor P-TEFb from its repressor, the 7SK RNA [9, 10]. If hnRNP A1 and A2 proteins normally participate in the release of P-TEFb from 7SK, decreasing the level of hnRNP A1 and A2 should increase the association of P-TEFb with 7SK. To test this prediction, we carried out immunoprecipitation assays using HCT116 cells and antibodies directed against CDK9, and then measured by quantitative RT-PCR the level of 7SK RNA associating with CDK9. The immunoprecipitation assay shows that DRB treatment is associated with a loss of the CDK9/7SK interaction (Fig 4A), despite more 7SK RNA being made (Fig 4B). This result is consistent with studies indicating that DRB promotes the accumulation of CDK9 at sites of stalled transcription [9, 34, 48]. In contrast, depleting A1/A2 significantly increased the association of CDK9 with the repressor 7SK RNA (Fig 4A). The amount of 7SK associating with CDK9 increased by 20 percentage points, a value normalized for the amount of 7SK present in each sample. The amount of CDK9 in cells treated with siA1/A2 did not change (Fig 4C). These results therefore suggest that the RNAi-mediated knockdown of A1/A2 may prevent the efficient dissociation of CDK9 from 7SK, and this may result in a transcription elongation defect. Although the mechanisms by which DRB and the knockdown of A1/A2 affect CDK9 activity may be different (CDK9 enzymatic inhibition versus association of CDK9 with the repressor 7SK, respectively), ultimately the impact on transcription may be similar.

### hnRNP A1/A2 proteins affect the distribution of RNA polymerase II on the *Kitlg* gene

Our model postulates that the increased association of pol II with the mycUP1 gene when A1/A2 are depleted results from a problem in transcription elongation affecting many P-TEFb-dependent genes. Expression and ChIP-PCR assays (Fig 3B–3E) suggest that *Kitlg* belongs to this latter class and that it may have experienced such an elongation defect. The impact of DRB on pol II binding to *Kitlg* is indicative of promoter-proximal stalling (Fig 3C). For sorbitol, while a decrease in pol II occupancy in the gene body of *Kitlg* was seen 8 hours post-treatment, the situation at promoter-proximal positions indicated a slight increase at position +254 and a slight decrease at position +854 (Fig 3D). Monitoring pol II occupancy on *Kitlg* at earlier times (Fig D, panels A and C in S1 File) indicated a drop in occupancy after 1 hour, followed by restoration of pol II occupancy only at positions +254 and +854, consistent with defective P-TEFb activity. In contrast, no change in pol II occupancy was observed on the mycUP1 gene 1 hour post-treatment, but occupancy increased afterwards (Fig D, panels B and C in S1 File), indicating that the increase in pol II occupancy on mycUP1 might be occurring only after the initial drop in pol II occupancy on *Kitlg* is detected.

The impact of depleting A1/A2 on pol II occupancy on *Kitlg* was not as clear (Fig 3E), possibly reflecting the weaker effect of siA1/A2 on *Kitlg* expression (Fig 3B). With the hope of improving sensitivity, we performed a pol II-ChIP assay followed by deep sequencing. The ChIP-seq data confirmed that both DRB and the RNAi-mediated knockdown of A1/A2 stimulated RNA pol II occupancy on *Egr1* (Fig D, panel D in S1 File). For *Kitlg*, the decreased expression imposed by DRB was associated with a redistribution of pol II at more downstream positions, suggesting promoter-proximal pausing (boxed region in Fig D, panel E in S1 File). The depletion of A1/A2 also provoked a slight redistribution in pol II occupancy in favor of more downstream positions, in addition to an overall decrease in pol II occupancy (Fig D, panel E in S1 File). Thus, these results suggest that the siRNA-mediated depletion of A1/A2 alters the promoter-proximal occupancy of pol II on *Kitlg*.

### The siRNA-mediated depletion of A1 and A2 modifies the rate of elongation

**Fig 5:**
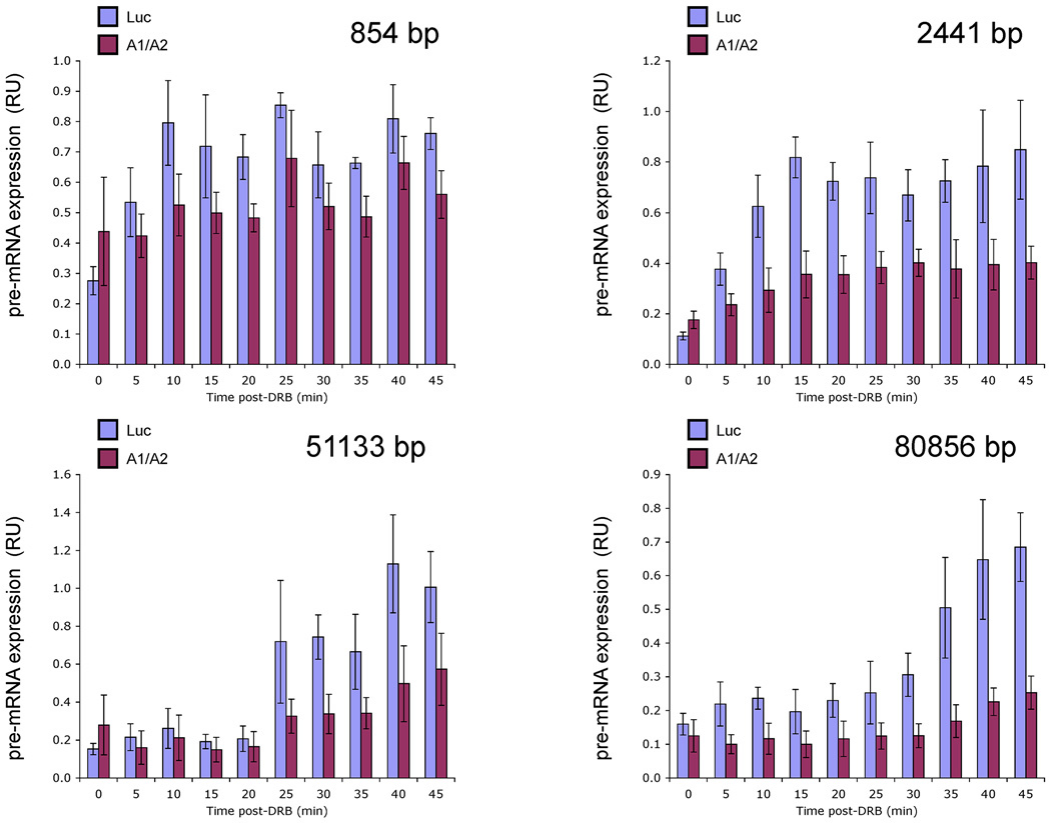
Transcription elongation recovery assay. Change in the rate of transcription of different portions of *Kitlg* upon depletion of A1 and A2 by RNAi. Seventy-two hours after transfecting a control siRNA against *luciferase* (Luc) or siA1_6_ + siA2_1_ (A1/A2) in HCT116 cells (H55-34 clone), DRB was applied, washed out, and the recovery of transcription was assessed at different positions. Each graph illustrates the position analyzed and the level of RNA at each time after the DRB block was released. The graphs represent averages of three independent experiments and standard deviations are provided. The positions analyzed on *Kitlg* are shown schematically in the map provided in Fig 3C.

To evaluate more directly if low levels of A1/A2 elicit an elongation defect on *Kitlg*, we relied on a quantitative RT-PCR-based elongation rate assay that measures the recovery of transcription at different positions of a gene following the release of an elongation block [41]. Under transfected conditions with a control siRNA (luc), transcription signals reappear gradually with time throughout *Kitlg* yielding an elongation rate of approximately 2.3 Kbp/min (blue histograms in Fig 5), consistent with previous values [41]. Upon releasing the elongation block in A1/A2-depleted cells, transcription output at the proximal position +854 closely followed that of the control transfection (Fig 5, red histograms in top left panel). In contrast however, the recovery of transcription at all downstream positions (other panels in Fig 5) was significantly reduced compared to the control situation, consistent with impaired elongation in A1/A2-depleted cells.

### The siRNA-mediated depletion of hnRNP A1/A2 has a broad impact on gene expression and polymerase II occupancy

**Fig 6:**
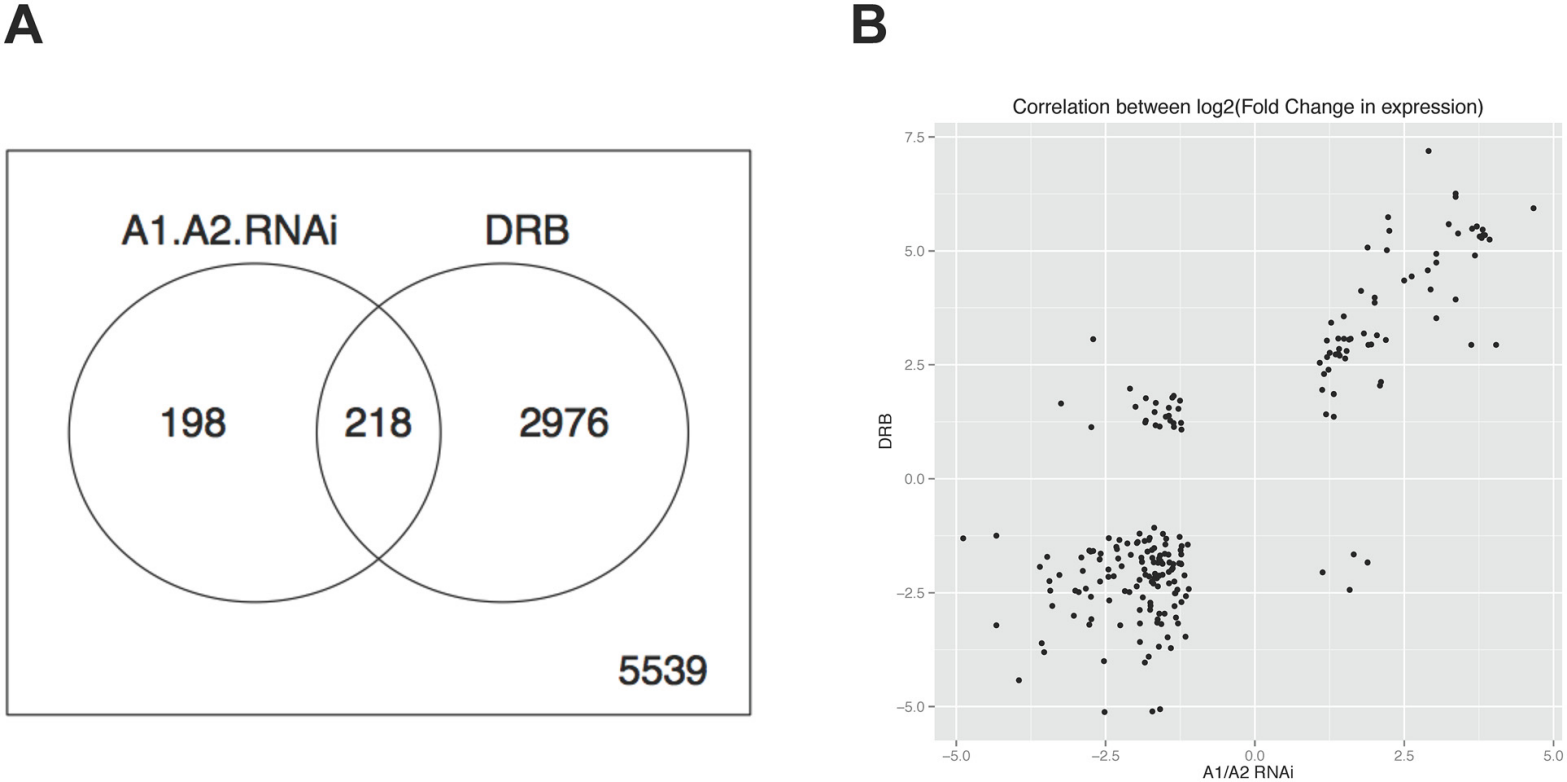
Impact of A1/A2 depletion and DRB on gene expression. **A**, The transcriptome of untreated (NS), A1/A2-depleted by RNAi and DRB-treated HCT116 cells was sequenced and reads were assigned. The Venn diagram depicts the number of genes in each sample that display a difference in expression relative to the NS sample, and only includes genes displaying a log2 (fold change) ≥ 2 with a correction for genes expressed at lower level using the following equation: corrected-log2(fold change) = log2(fold change) + 1/gamma^(exp-exp.offset), where gamma = 5 and exp.offset = 0, exp is the average expression level expressed as base 10 log, and fold change is the measured fold change in expression. The *p*-value of the DRB and ΔA1/A2 overlap is 1.4e-12 (Fisher’s exact test). **B**, Correlation map plotting the expression changes for the overlapping set of reactive genes in the DRB-treated and siA1/A2-depleted samples. Only genes with fold changes ≥ 2 are considered. The correlation coefficient is 0.692 (Pearson correlation ^2).

To assess how generalized the knockdown of A1/A2 affects gene expression, we carried out next-generation transcriptome sequencing using untreated, A1/A2-depleted and DRB-treated HCT116 cells (H55-34 clone). The read coverage for the complete set of genes is available at GEO accession number GSE67617. When considering ≥2-fold changes in expression relative to the untreated sample, the analysis revealed that more than half of the genes affected by the depletion of A1/A2 were also affected by DRB (*p* = 1.4e-12 using Fisher exact test; Fig 6A). Although a gene annotation analysis did not reveal enrichment for specific pathways for genes in this overlap, the response of these genes shows a strong correlation (R^2^ = 0.692; Fig 6B), as would be expected if the majority of the changes elicited by DRB and the RNAi-mediated knockdown of A1/A2 is caused by a common defect.

**Fig 7:**
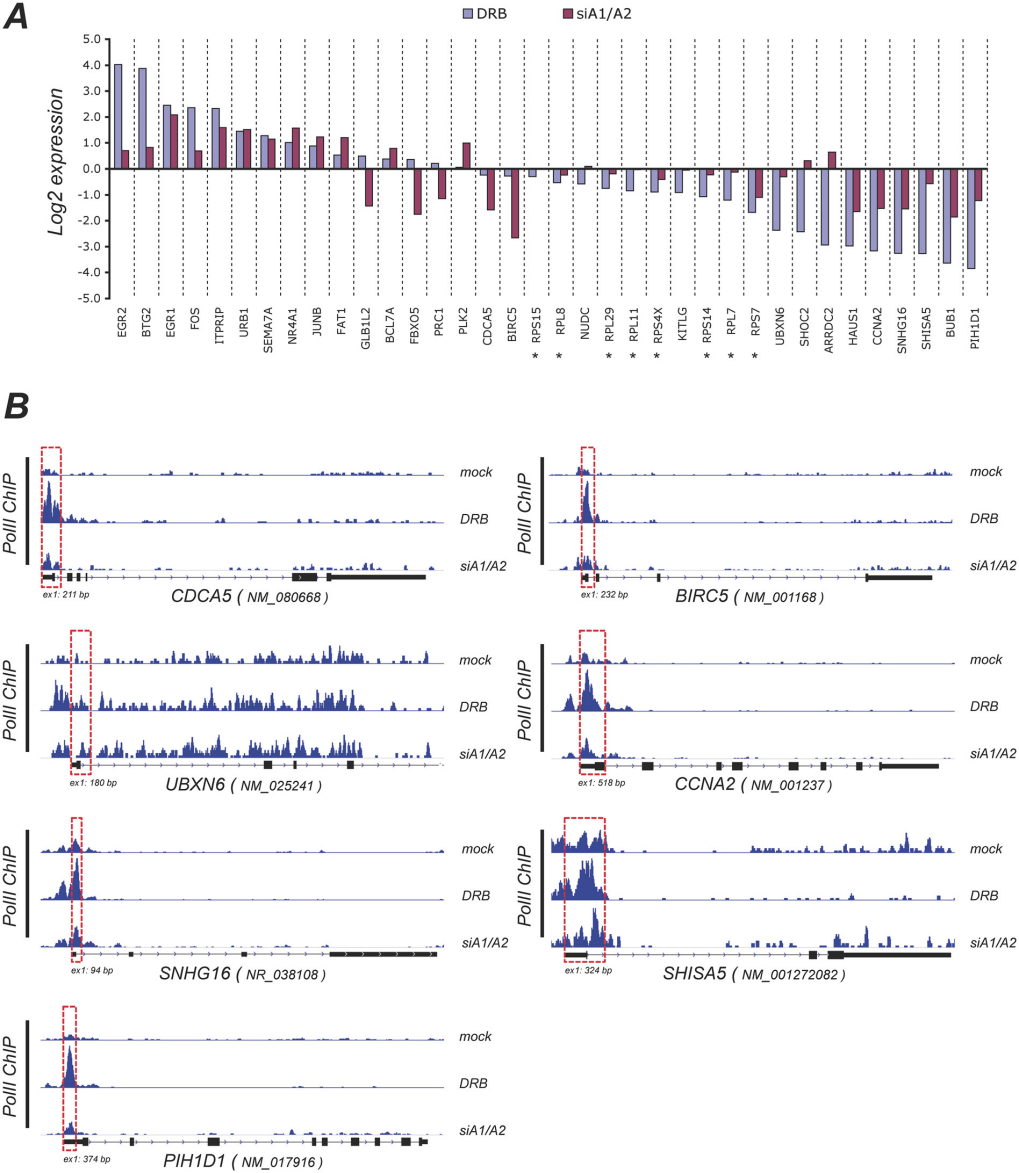
Changes in expression and RNA polymerase II occupancy elicited by DRB and the siRNA-mediated depletion of A1/A2. **A**, Histograms depicting the difference in expression for selected genes based on reads assignment and normalized for library sizes using TMM normalization. Genes encoding ribosomal proteins are indicated with an asterisk. **B**, Density of ChIP-seq reads for RNA polymerase II (H-224 antibody) on selected genes (with the major RefSeq transcripts indicated) in mock-treated, DRB-treated and siA1/A2-treated samples. Genes in panel B are transcribed from left to right. The size in bp of the first exon is indicated. Red dashed boxes indicate the promoter-proximal regions displaying an increase in pol II occupancy upon treatment with DRB and the siRNA-mediated knockdown of A1/A2.

**Fig 8:**
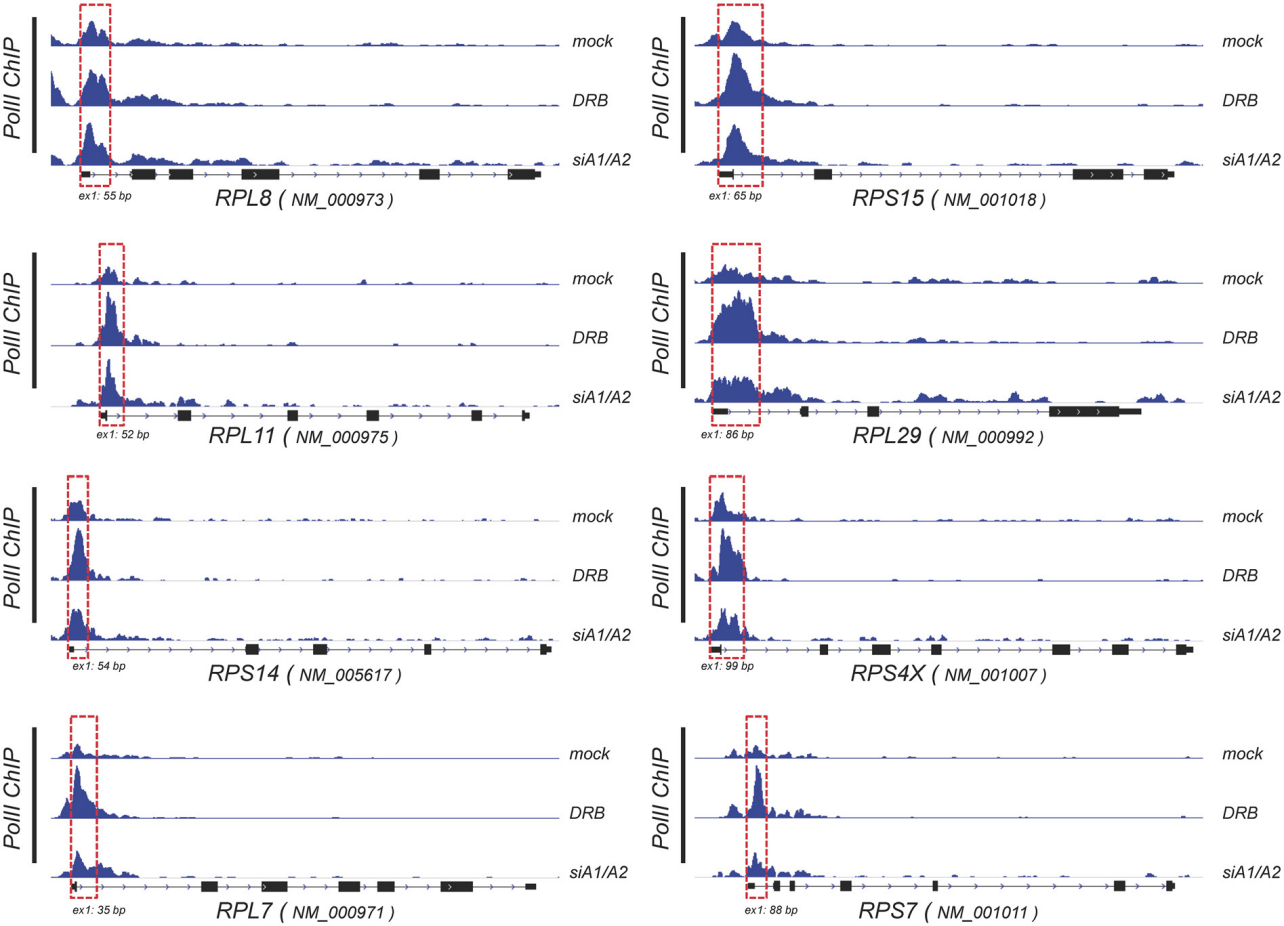
Changes in RNA polymerase II occupancy on ribosomal protein-encoding genes elicited by DRB and the depletion of A1/A2. Density of ChIP-seq reads for RNA polymerase II (H-224 antibody) on genes encoding ribosomal proteins (major RefSeq transcripts indicated). Genes are transcribed from left to right. The size in bp of the first exon is indicated. Red dashed boxes indicate the promoter-proximal regions displaying an increase in pol II occupancy upon treatment with DRB and the siRNA-mediated knockdown of A1/A2.

Fig 7A displays a selection of genes with a variety of responses in amplitude and direction following the siRNA-mediated depletion of A1/A2 and the treatment with DRB. DRB and the siRNA-mediated depletion of A1/A2 proteins promote both increases and decreases in gene expression. Although the source of the defect in P-TEFb activity is different for each treatment (inhibition of CDK9 activity for DRB, and preventing the release of P-TEFb from 7SK for the siRNA-mediated drop in A1/A2), they should both elicit a promoter-proximal accumulation of pol II on genes that are repressed. Accordingly, an analysis of the pol II-ChIP-seq data reveals that several genes whose expression was repressed by both treatments, including a subset encoding ribosomal proteins, displayed an increase in pol II occupancy at promoter-proximal positions (Figs 7B and 8). In general, the amplitude of change was superior for DRB than for the siRNA-mediated knockdown of A1/A2. In all cases, pol II accumulation began at, or very near, transcription initiation sites and extended downstream to 175 bp for *SNHG16*, *PIH1D1*, *BIRC5*, and most ribosomal protein genes, and up to 500 bp for *UBXN6*, *CCNA2* and *SHISA5* (see dashed boxes in Figs 7B and 8). These promoter-proximal increases in pol II occupancy in genes repressed by DRB and by the siRNA-mediated knockdown of A1/A2 suggest polymerase II pausing.

## Discussion

We provide evidence in support of a role for hnRNP A1 and A2 proteins in the control of transcription elongation by RNA polymerase II. Our study was triggered by noting that the expression of a mycUP1 reporter gene was strongly stimulated when the A1 and A2 proteins were depleted by RNA interference, or when the transport of these proteins to the nucleus was compromised following osmotic shock with sorbitol. The increase in the steady-state level of mycUP1 transcripts provoked by these treatments was abrogated by the transcription inhibitor actinomycin D, suggesting that the increase was caused by transcription stimulation rather than by improved transcript stability. Consistent with the transcription stimulation model, we observed a strong increase in RNA polymerase II occupancy on the body of the mycUP1 reporter gene when cells were depleted of nuclear A1/A2.

The increase in mycUP1 RNA induced by sorbitol occurred even when protein synthesis was inhibited, suggesting that A1/A2 may have a direct impact on transcription. A possible link between A1 and transcription elongation had been suggested previously based on *in vitro* assays indicating that A1 could prevent the interaction of the transcription elongation factor P-TEFb to its repressor molecule, the 7SK RNA [9, 10]. Since expression of mycUP1 was also stimulated by DRB, a specific inhibitor of CDK9 that is component of the transcription elongation factor P-TEFb, the relationship between A1/A2 and P-TEFb was investigated in more detail. Importantly, and consistent with previous *in vitro* reports [9, 10], we found that the RNAi-mediated knockdown of hnRNP A1/A2 increased the association of CDK9 with 7SK RNA. The SR protein SRSF2 has been shown to interact with 7SK RNA at paused polymerase sites where it mediates the release of active P-TEFb when SRSF2 binding is transferred to nascent promoter-proximal transcripts and, possibly, exonic splicing enhancers [49]. Because SRSF2 and hnRNP A1/A2 can both interact with the loop 3 region of 7SK RNA [10, 49], the interaction of hnRNP A1/A2 to 7SK may occur at this stage to favor the dissociation of SRSF2, and hence contribute to the release of active P-TEFb. Although the individual knockdown of hnRNP A1 or B (A2) proteins did not affect the progression of RNA polymerase II in the SRSF2 study [49], our results indicate that the depletion of both A1 and A2 was required to overcome compensatory mechanisms regulating their expression (see Fig 1).

If reducing the level of A1/A2 compromises the release of P-TEFb from 7SK, this should in turn elicit promoter-proximal RNA polymerase II pausing. Consistent with this prediction, the results of ChIP-PCR and ChIP-seq assays indicated that DRB and the siRNA-mediated depletion of A1/A2 increased the occupancy of pol II at promoter-proximal positions. Moreover, this depletion was associated with an elongation rate defect in *Kitlg*. Notably, an analysis of the transcriptome of A1/A2-depleted and DRB-treated cells revealed that more than 50% of genes affected by the A1/A2 depletion (218 out of 416 genes) were also affected by DRB. For many of the genes that displayed reduced expression, including several genes encoding ribosomal proteins, both DRB and the siRNA-mediated depletion of A1/A2 increased promoter-proximal pol II occupancy. Thus, our results support the existence of a functional link between A1/A2 and P-TEFb, and are consistent with the view that A1/A2 levels impact P-TEFb activity.

**Fig 9:**
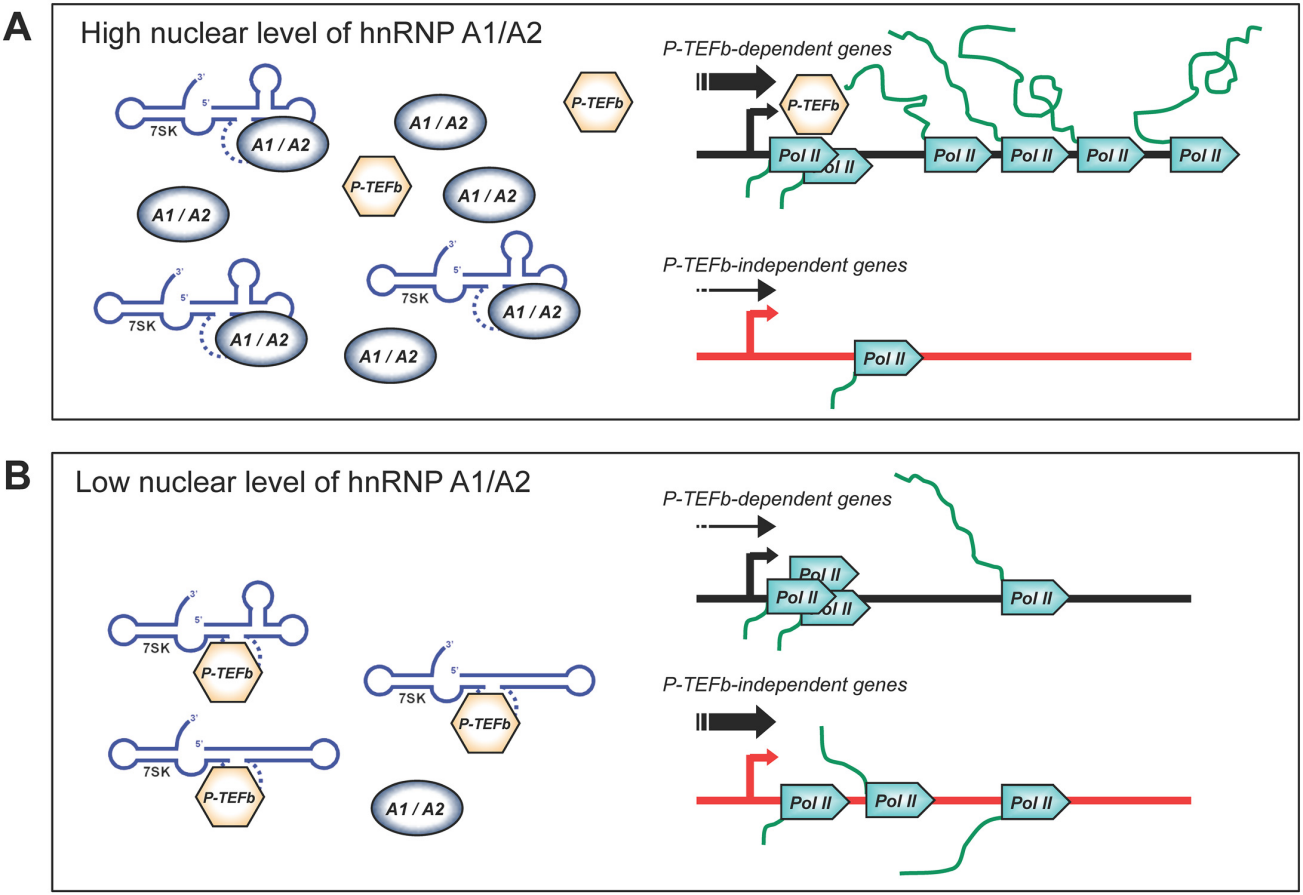
Model to explain how a decrease in hnRNP A1/A2 proteins reduces transcription elongation of P-TEFb-dependent genes but stimulates expression of P-TEFb-independent genes. In panel A, normally high levels of nuclear hnRNP A1/A2 proteins ensure the release of P-TEFb from the repressor 7SK RNP which is used for the efficient transcription of P-TEFb-dependent genes, such as *Kitlg* and many genes encoding ribosomal proteins. A reduction in the levels of nuclear hnRNP A1/A2 proteins (panel B) would prevent the efficient release of P-TEFb from 7SK, eliciting promoter-proximal pausing of RNA polymerase II on P-TEFb-dependent genes, and a decrease in their expression. The stalling of polymerases at promoter-proximal locations would reduce the average number of polymerases associating with P-TEFb-dependent genes, thereby increasing the level of free polymerases which would then associate preferentially with, and stimulate the expression of, P-TEFb-independent genes, such as the mycUP1 reporter gene and *Egr1*.

A drop in the transcription of P-TEFb-dependent genes that are normally expressed at high levels may impact the expression of genes that do not normally require P-TEFb. One possible scenario through which this can occur would be that polymerases that complete their round of transcription and join the free pool may be loaded preferentially on genes that have no paused polymerases build up at promoter-proximal positions (Fig 9). This situation may explain why the transcription of genes such as the reporter mycUP1 gene and *Egr1* was stimulated by the RNAi-mediated knockdown of A1/A2, by osmotic shock and by DRB.

Future studies will be required to identify the parameters that make some genes respond to both a drop A1/A2 and to DRB, while others respond to DRB but not to the depletion of A1/A2 proteins. This distinct behavior may point to the existence of a subset of P-TEFb-dependent genes that use A1/A2 to recruit and activate P-TEFb. Alternatively, the observation that not all DRB-responsive genes responded to a partial depletion of A1/A2 may reflect the existence of genes with a lower threshold of sensitivity that is only reached by using DRB or by having a more complete depletion in the level of nuclear A1/A2 proteins. Thus, it is for the moment unclear if the contribution of A1/A2 is a feature of all P-TEFb-dependent genes or is restricted to a subset of them. Notwithstanding, given the role of A1/A2 in splicing and alternative splicing [45, 46, 50], it makes biological sense to couple the level of splicing factors with early events in transcription elongation, since the transcription of genes with introns and alternative exons whose splicing is regulated by A1/A2 would occur efficiently only when the A1/A2 proteins are abundantly expressed. It is also unclear how the release of P-TEFb from 7SK could be linked co-transcriptionally with pre-mRNA maturation events that may be occurring farther downstream in transcription units. Possibly, some of the genes that use A1/A2 for splicing may have acquired specific promoter or chromatin features that link their transcription elongation rates with the recruitment of P-TEFb through A1/A2-dependent 7SK remodeling.

There is increasing evidence to indicate that the release of RNA polymerase II from its promoter-proximal position is an important mechanism of gene activation and a common strategy to control differential gene expression [51]. Moreover, destabilizing the 7SK RNA in the zebrafish causes severe developmental defects [52]. If the levels of A1/A2 proteins can alter the expression of important regulatory genes such as *Kitlg* and genes encoding ribosomal proteins by modulating the release of P-TEFb from the 7SK complex, tissue-specific variations in hnRNP A1/A2 proteins may have a broad impact on transcriptional and developmental regulation.

## Supporting Information

S1 File **Fig. A. mycUP1 expression is stimulated by the depletion of nuclear hnRNP A1 and A2 in WI38VA13 cells**. Western analysis was used to test the effects of sorbitol and RNAi targeting of A1 and A2 in a WI38VA13 clone stably expressing mycUP1. **Fig. B. Examples of sqRT-PCR results for RNAi and sorbitol treatments**. Dilution of a control cDNA sample was used to evaluate the fold difference using Quantity One software (Bio-Rad). **Fig. C. mycUP1 expression is stimulated by sorbitol, even in the presence of cycloheximide**. Quantification of duplicate experiment testing the impact of cycloheximide on the stimulation of mycUP1 elicited by sorbitol. HCT116 cells expressing mycUP1 were treated with 1 or 10 μg/ml of cycloheximide 1 hour prior to sorbitol treatment (1 hour). RNA was collected 24 hours later. Histograms show a log scale quantification of mycUP1 RNA levels (sqRT-PCR) that have been normalized to β-actin RNA levels. **Fig. D. Chromatin immunoprecipitation assays using anti-RNA polymerase II antibody. A**, ChIP-PCR assays were performed to monitor time-dependent changes of pol II occupancy on the *Kitlg* gene following sorbitol treatment. **B**, ChIP assays were performed to monitor time-dependent changes in pol II occupancy on the mycUP1 gene following sorbitol treatment. The numbers indicate the position of the center of the amplicon relative to the transcription start site (see Fig 3). **C**, The values for specific positions in *Kitlg* and mycUP1 from panels A and B were plotted relative to time to illustrate that stimulation of mycUP1 transcription is detected after 2 hours while the drop in *Kitlg* expression is detected after 1 hour. **D**, Density of ChIP-seq reads for RNA polymerase II (H-224 antibody) on *Egr1* (major RefSeq transcript indicated) in mock-treated, DRB-treated and siA1/A2-treated samples. **E**, Density of Pol II ChIP-seq reads on *Kitlg* in mock-treated, DRB-treated and siA1/A2-treated samples Only the promoter-proximal region of the gene is shown. The boxed portion represents the region of the *Kitlg* gene where an increase in pol II occupancy is noted in DRB-treated and A1/A2-depleted samples. **Table A. Sequences of siRNAs. Table B. Oligonucleotides used for RT-PCR analysis. Table C. Oligonucleotides used for the qPCR time-course analysis of transcription and for chromatin immunoprecipitation assays**.
(PDF)
