# Supplementary material for: A Function for the hnRNP A1/A2 Proteins in Transcription Elongation
Source: PLoS One. 2015 May 26;10(5):e0126654. doi: 10.1371/journal.pone.0126654 (PMC4444011; doi:10.1371/journal.pone.0126654)
Supplement: S1 File — Fig. A. mycUP1 expression is stimulated by the depletion of nuclear hnRNP A1 and A2 in WI38VA13 cells. Western analysis was used to test the effects of sorbitol and RNAi targeting of A1 and A2 in a WI38VA13 clone stably expressing mycUP1. Fig. B. Examples of sqRT-PCR results for RNAi and sorbitol treatments. Dilution of a control cDNA sample was used to evaluate the fold difference using Quantity One software (Bio-Rad). Fig. C. mycUP1 expression is stimulated by sorbitol, even in the presence of cycloheximide. Quantification of duplicate experiment testing the impact of cycloheximide on the stimulation of mycUP1 elicited by sorbitol. HCT116 cells expressing mycUP1 were treated with 1 or 10 μg/ml of cycloheximide 1 hour prior to sorbitol treatment (1 hour). RNA was collected 24 hours later. Histograms show a log scale quantification of mycUP1 RNA levels (sqRT-PCR) that have been normalized to β-actin RNA levels. Fig. D. Chromatin immunoprecipitation assays using anti-RNA polymerase II antibody. A, ChIP-PCR assays were performed to monitor time-dependent changes of pol II occupancy on the Kitlg gene following sorbitol treatment. B, ChIP assays were performed to monitor time-dependent changes in pol II occupancy on the mycUP1 gene following sorbitol treatment. The numbers indicate the position of the center of the amplicon relative to the transcription start site (see Fig 3). C, The values for specific positions in Kitlg and mycUP1 from panels A and B were plotted relative to time to illustrate that stimulation of mycUP1 transcription is detected after 2 hours while the drop in Kitlg expression is detected after 1 hour. D, Density of ChIP-seq reads for RNA polymerase II (H-224 antibody) on Egr1 (major RefSeq transcript indicated) in mock-treated, DRB-treated and siA1/A2-treated samples. E, Density of Pol II ChIP-seq reads on Kitlg in mock-treated, DRB-treated and siA1/A2-treated samples Only the promoter-proximal region of the gene is shown. The boxed portion represent [file pone.0126654.s001.pdf]

Figure A

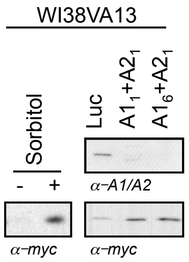

Figure B

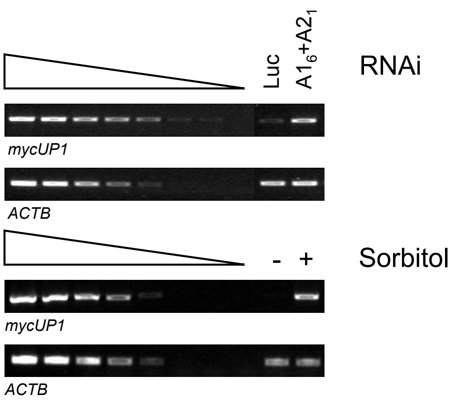

Figure C

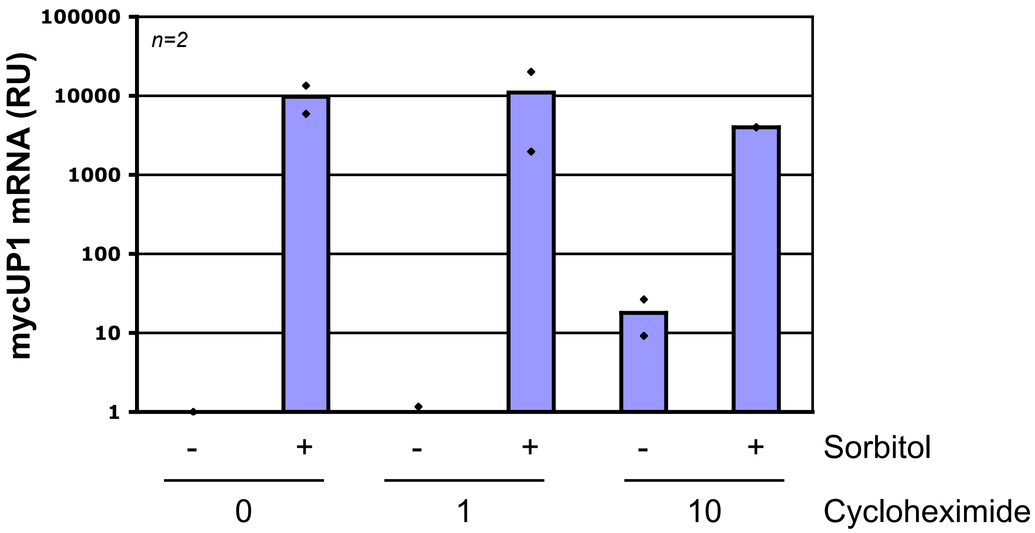

n=2
mycUP1 mRNA (RU)
100000
10000
1000
100
10
1
- + - + - +
Sorbitol
0
1
10
Cycloheximide

Figure D

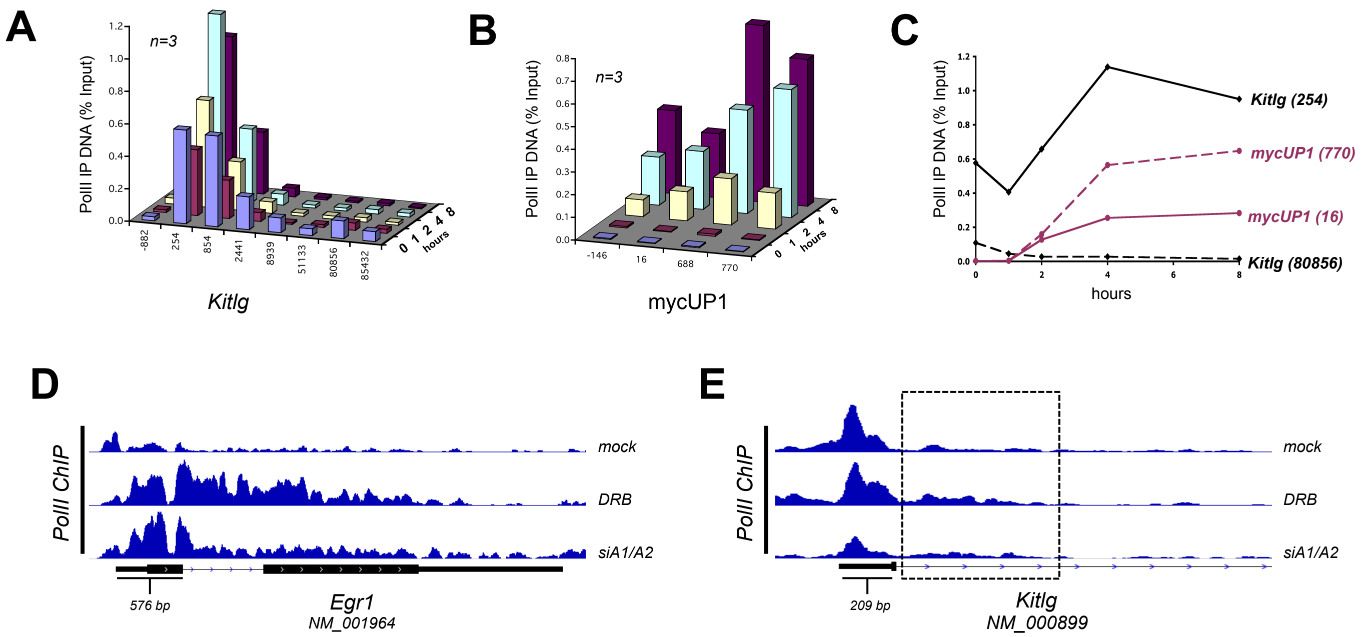

A
n=3
PolII IP DNA (% Input)
Kitlg
-882
254
854
2441
8939
51133
80856
85432
0 1 2 4 8
hours
B
n=3
PolII IP DNA (% Input)
mycUP1
-146
16
688
770
C
PolII IP DNA (% Input)
Kitlg (254)
mycUP1 (770)
mycUP1 (16)
Kitlg (80856)
hours
D
PolII ChIP
mock
DRB
siA1/A2
576 bp
Egr1
NM_001964
E
PolII ChIP
mock
DRB
siA1/A2
209 bp
Kitlg
NM_000899

Table A. Sequences of siRNAs.

Gene target	siRNA name	SEQUENCE
hnRNP-A1	$A1_1$	UGGGGAACGCUCACGGACU
hnRNP-A1	$A1_2$	UGAGAGAUCCAAACACCAA
hnRNP-A1	$A1_5$	AUCAUGACUGACCGAGGCA
hnRNP-A1	$A1_6$	CUUUGGUGGUGGUCGUGGA
hnRNP-A2	$A2_1$	GCUUUGAAACCACAGAAGA
luciferase	luc	AACAUCACGUACGCGGAAUAC

Table B. Oligonucleotides used for RT-PCR analysis.

RT Reaction

RT101	GAGGACTCGAGCTCAAGCTTTTTTTTTTTTTTTTTTTTVN
RT102_7SK	GAGGACTCGAGCTCAAGCGCCTCATTTGGATGTGTCTG

	Forward		Reverse	
**sqRT-PCR**				
ACTB	ACTB-F	GCGGGAAATCGTGCGTGACATT	ACTB-R	GATGGAGTTGAAGGTAGTTTCGTG
CMVmycUP1	UP1-F	CAAGAGAAGATTCTCAGC	RT102	GAGGACTCGAGCTCAAGC
**qRT-PCR (fig 2)**				
ACTB	ACTB_gbl_for_1	TTCCTGGGCATGGAGTC	ACTB_gbl_rev_1	CAGGTCTTTGCGGATGTC
CMVmycUP1	pcDNA-F1	CTGCAGATATCCAGCACAGT	RT102	GAGGACTCGAGCTCAAGC
**qRT-PCR**				
mycUP1	pcDNA-F1	CTGCAGATATCCAGCACAGT	RT102	GAGGACTCGAGCTCAAGC
EGR1	qRT EGR1-F4	TGGGTGCCGCTGAGTAAATG	qRT EGR1-R4	CTGACCGCAGAGTCTTTTCCTG
KITLG	qRT KITLG-F1	CAAAAATCCCCCTGGAGAC	qRT KITLG-R1	CAACTGCCCTTGTAAGACTTGG
7SK (1)	qRT 7SK-F1	GAGGGCGATCTGGCTGCGACAT	qRT 7SK-R1	ACATGGAGCGGTGAGGGAGGAA
7SK (2)	qRT 7SK-F2	AGGACCGGTCTTCGGTCAA	qRT 7SK-R2	TCATTTGGATGTGTCTGGAGTCT
7SK (3)	qRT 7SK-F3	CATCCCCGATAGAGGAGGAC	RT102	GAGGACTCGAGCTCAAGC
ACTB	qRT ACTB-F1	GCGGGAAATCGTGCGTGACATT	qRT ACTB-R1	GATGGAGTTGAAGGTAGTTTCGTG
GAPDH	qRT GAPDH F2	TGCACCACCAACTGCTTAGC	qRT GAPDH-R2	GGCATGGACTGTGGTCATGAG
B2M	qRT B2M-F1	TGCTGTCTCCATGTTTGATGTATCT	qRT B2M-R1	TCTCTGCTCCCCACCTCTAAGT
RPL13A	qRT RPL13A-F1	CCTGGAGGAGAAGAGGAAAGAGA	qRT RPL13A-R1	TTGAGGACCTCTGTGTATTTGTCAA

Table C. Oligonucleotides used for qPCR time-course analysis of transcription and for chromatin immunoprecipitation assays.

Gene	Amplicon ID	Forward Primer	Reverse Primer
mycUP1	-146	ATGGTGATGCGGTTTTGGCAG	AAGTCCCGTTGATTTTGGTGCC
mycUP1	16	ATTGACGCAAATGGGCGGTAG	TTTAAACGCTAGCCAGCTTGGG
mycUP1	688	GATGACCATGACTCTGTGGA	TCTGCAGAATTCTTAGCGAC
mycUP1	770	GAAGCAAGAGATGGCTAGTG	AACTAGAAGGCACAGTCGAG
Egr1	-1625	TCCTTCCCCACATTCTCTTTCTGC	ACCCCAAAGGGAACAGGGATTC
Egr1	-807	ACAGTGTCCCAAGAACCAAGTG	TCGATCTATGGCACGGTGTCTTTC
Egr1	-65	TGCCATATTAGGGCTTCCTGCTTC	ATTTGAAGGGTCTGGAACGGC
Egr1	2224	AAGTTTGCCAGGAGCGATGAAC	AGGCCACAACACTTTTGTCTGC
Egr1	3443	TTGGCCAACAATCCTTTCTGCC	TTGGACATGGCTGTTTCAGGC
Kitlg	-882	TGAGAAAGTGAGAACGCAGTCC	AAACCTGTAATCAGGGCGAAGC
Kitlg	254	TAATCTGCCAAACTTCTGGGGC	GAGAAGCAGGCAAGGGCG
Kitlg	854	AGCCAGTGTCAAGTTTGGAGC	AACATTCCATGGCCCAAAAGCC
Kitlg	2441	TGCTGCCGACCAGAGAAATTTG	AGGGCCTGCACTTTAACTTGTG
Kitlg	8939	TTCACAGCACTTTTCCGCTGAG	AATGCGCTGTGGTTAATGCCAG
Kitlg	51133	TGTTCGTTTCTGCGAAGACCAG	TTTTTGGCACCCATTCCCACTC
Kitlg	80856	TGGGGCTTAAATGCCTTGTGTC	ACCTCAGCTTTGATTGTCACCC
Kitlg	85432	AACCCAGGTGCTTTGAGAAGC	ACAGTCAATGCCACACACTGAG

The amplicon ID indicates the position of the central base pair of the amplicon relative to the transcription start site based on Aceview database for the RefSeq NM_000899 for Kitlg.
